# Easily Missed: A Case Series of New Heart Failure in Young Adults

**DOI:** 10.1155/2020/7251609

**Published:** 2020-08-23

**Authors:** Eric J. Hall, Jonathan Menachem, Lynne Warner Stevenson, Jessica Huston

**Affiliations:** ^1^Department of Medicine, Vanderbilt University Medical Center, Nashville, TN, USA; ^2^Division of Cardiovascular Medicine, Vanderbilt University Medical Center, Nashville, TN, USA

## Abstract

*Background*. While uncommon, heart failure (HF) can present in young adults from a variety of causes. Identifying HF in a young patient presents many challenges, the foremost of which is recognition of the signs and symptoms of HF. *Case Summary*. We present four cases of new diagnosis of HF (due to familial cardiomyopathy, tachycardia-induced cardiomyopathy, spontaneous coronary artery dissection, and peripartum cardiomyopathy) to highlight the range of etiologies and presentations requiring recognition in this patient population. *Discussion*. A high index of suspicion is needed to diagnose HF in young adults, who may not present with classic signs and symptoms. Young adults represent a unique patient population that differs from the older patients with HF. Young adults with newly diagnosed HF should be promptly referred to a center offering full diagnostic capabilities and advanced cardiac therapies.

## 1. Introduction

Heart failure in young adults is an uncommon yet potentially fatal diagnosis which, despite increasing incidence, is often initially misdiagnosed in favor of more common diagnoses of viral infections or pulmonary disease [1, 2]. Little data exist regarding this unique patient population [3]. To highlight the challenge and diversity of causes of heart failure in young adults, we discuss four cases of newly diagnosed systolic heart failure (HF).

## 2. Case Presentations

### 2.1. Case 1: Hereditary Nonischemic Cardiomyopathy

A previously healthy 26-year-old South Asian man presented to a local emergency room with two months of dyspnea and lower extremity edema. Several primary care and emergency providers had evaluated him for shortness of breath, cough, and fatigue over several months prior to this presentation. Despite multiple courses of antibiotics, proton-pump inhibitors, and antihistamines, his symptoms had progressed. His family history included his father who was diagnosed in his 20s with HF which led to his death at age 34. Eventually, worsening leg edema and resting dyspnea led to an echocardiogram, which revealed severe biventricular dysfunction (left ventricular ejection fraction (LVEF) of 10%) and a 7 cm left ventricular (LV) thrombus. Left heart catheterization at the referring center showed normal coronary arteries but elevated filling pressures and low cardiac output, prompting the placement of an intra-aortic balloon pump (IABP) prior to transfer to our institution.

On arrival, physical exam demonstrated generalized edema. The patient developed anuric renal failure and required high-dose inotropes despite IABP. He was cannulated for venoarterial extracorporeal membrane oxygenation (VA-ECMO), and subsequently, an atrial septostomy was performed for LV unloading in the setting of LV thrombus. He was listed 1A for transplant and successfully transplanted. Subsequent genetic testing revealed a pathologic *FLNC* mutation (R650X, resulting in protein truncation). He is currently two years posttransplant.

### 2.2. Case 2: Thyrotoxicosis-Associated Tachycardia-Mediated Cardiomyopathy

A 37-year-old Black woman with a history of asthma presented to a local emergency department with two weeks of nonproductive cough, palpitations, weight gain (32 lbs), and severe dyspnea on exertion. Her family history included a father, grandfather, and cousin with a history of HF.

Pertinent labs on arrival to the outside hospital included an undetectably low TSH and significant elevated pro-BNP. EKG revealed atrial fibrillation with rapid ventricular rate. For this, she received IV dexamethasone for potential thyroid storm with diltiazem and propranolol to control her heart rate. Although initially alert and normotensive, she became progressively lethargic and hypotensive, requiring intubation and vasopressors prior to transfer.

On arrival, she was hypotensive and groggy. Echocardiography revealed severe global LV hypokinesis and moderately depressed right ventricular (RV) function. Unresponsive to vasopressors, she underwent emergent cannulation for VA-ECMO. She was stabilized on VA-ECMO for 10 days, during which she was treated for thyroid storm and diagnosed with Grave's disease. Repeat echocardiogram two weeks later showed recovery of ventricular function to LVEF 55–60% and mildly reduced RV function. She was discharged on guideline-directed medications for systolic HF and had NYHA class II symptoms five months later. Genetic testing was declined by the patient.

### 2.3. Case 3: Acute Heart Failure Secondary to Spontaneous Coronary Artery Dissection

A previously healthy 35-year-old white man without family history of cardiac disease presented to an outside hospital twice with shortness of breath during a month. He was prescribed repeated courses of antibiotics and steroids for presumed respiratory infection, but his symptoms persisted. On his third presentation, he was found to have an elevated troponin and lactate, with a chest X-ray consistent with pulmonary edema. Exam showed mild lower extremity edema with JVP unable to be visualized. He became hypotensive requiring inotropes and IABP placement. Echocardiogram revealed LVEF of 20% with global hypokinesis, moderate RV dysfunction, and an LV thrombus. He was transferred to our center for consideration of advanced therapies.

After arrival to our center, coronary angiography revealed a mid-LAD dissection with TIMI 2 flow. PET/CT demonstrated widespread myocardial scarring involving the anterior wall, septum, apex, and distal inferior wall, correlating with a large LAD infarct without viability. It was believed his RV dysfunction was secondary to persistently elevated left-sided filling pressures. Despite medical therapy and mechanical support, he remained persistently tachycardic, and intravenous inotropic therapy could not be weaned. A durable left ventricular assist device (LVAD) was placed 25 days after presentation. At three months postdischarge, he had NYHA class I symptoms with the LVAD in place. He is currently undergoing evaluation for heart transplantation.

### 2.4. Case 4: Peripartum Cardiomyopathy

Seven days after a spontaneous vaginal delivery, an otherwise healthy 19-year-old woman presented to an outside hospital with three days of nausea, cough, and dyspnea. Family history was negative for any cardiac disease or cardiovascular complications of pregnancy. On arrival, she was hypotensive, and echocardiography revealed LVEF 5–10% with mildly reduced RV function, concerning for peripartum cardiomyopathy. Despite stabilization with dobutamine and IV diuresis, she showed no signs of cardiac recovery. On hospital day 15, an LVAD was placed. Over the next 15 days, her cardiac function improved, and she was discharged on hospital day 32. Eleven months later, she had no resting symptoms and excellent exercise capacity without recurrence of symptoms. Genetic testing and preconception counseling for future pregnancies were discussed with the patient, who elected to defer this for the time being in the setting of anxiety.

## 3. Discussion

We describe four presentations of new systolic HF to emphasize the varied etiologies and urgency of diagnosis in young adult patients. The incidence of HF in young adults has increased for reasons which are not fully understood. Current estimates put the incidence of HF in young adults at between 0.02 and 1.00 per 1,000 person years [3]. Several studies have demonstrated increasing incidence of HF in young adults over the past several decades despite falling rates of HF overall [1, 4]. Notably, one of the largest studies of HF incidence by age found the incidence of HF in individuals <50 years old increased 50% between 1997 and 2012 (to 0.2 events per 1,000 person years among patients who are 35–44 years old) [4]. Idiopathic dilated cardiomyopathy is the most common cause of HF in young adults, although traditional risk factors (such as hypertension, obesity, and diabetes) also apply to young adults, which may in part drive the increasing incidence of HF in this age group [3]. Substance abuse (especially cocaine and anabolic steroid use) also represents an important risk factor which may play a role in the growing number of young adults with HF [3].

Despite the highly varied etiologies of HF in young adults, these patients can rapidly progress to end-stage HF and cardiogenic shock prior to diagnosis, as depicted in our cases. Given the acuity with which these patients can decompensate, early recognition of HF is essential. Our cases highlight several important lessons:

First, HF should be considered when a young adult presents with unexplained or refractory symptoms of shortness of breath. Among our four patients, all presented first to a primary care physician or an emergency department. These front-line clinicians face diminishing time to see increasing numbers of patients in the current state of the health care system. Despite the rising incidence of HF in the young, the pretest probability of HF in a young adult patient presenting with nonspecific complaints, such as difficulty breathing, remains quite low. Making the diagnosis is particularly challenging given young patients may not exhibit many of the classic signs of HF, such as lower extremity edema, which is more common in older patients and very rare in pediatric patients with HF [2]. Rales may not be present on the pulmonary exam if filling pressures are chronically elevated [5]. Third heart sounds are heard in fewer than half of patients [6]. The initial urgent examination may not include careful location of the jugular venous pulsations. These cases emphasize the importance of obtaining a thorough medical history including family history, as several of these patients had family members who developed HF at a young age. It should also be recognized that many illicit substances, known and unknown, can cause cardiomyopathy and may not be mentioned by patients.

For definitive therapy, young adults with a new diagnosis of HF should be promptly referred to a center which specializes in HF care and preferably one that can provide advanced therapies, including mechanical circulatory support and transplant. Some cases of acute HF in young adults can spiral rapidly into cardiogenic shock which can be fatal [7]. One-quarter of heart transplants (884 transplants) performed in 2018 in the United States took place in patients aged 18–49 years old, a reflection of the severity of illness these young patients can face [8]. The most common indication for heart transplant worldwide is nonischemic cardiomyopathy [9]. In addition to the nonischemic etiologies of HF highlighted in our cases, risk factors for ischemic heart disease are also increasing in young adults [3]. Prompt revascularization with stenting or bypass surgery may preserve cardiac function in patients with ischemic injury.

Finally, young adults may have a different appreciation of their health and their responsibilities to maintain it. In the CHARM study, young adults with HF self-reported worse quality of life despite having a higher average ejection fraction compared to older patients [2]. Young adults also demonstrated lower rates of medication adherence in the same study. Heart failure is more likely to occur at a young age in black patients than in other races [10]. In addition, young adults are more likely to experience idiopathic HF, which may be genetically-driven [11, 12]. Thus, genetic screening of family members, accompanied by genetic counseling, may be helpful in the long-term to guide patients and their families through the travails of a new and potentially unexpected diagnosis.

## 4. Conclusion

While an uncommon diagnosis, young adults can present with heart failure secondary to a variety of etiologies. A high index of suspicion should be maintained when young patients present with respiratory symptoms or fatigue which is more severe than expected for a typical seasonal viral illness or which persists despite conservative therapies. Prompt referral to a center capable of advanced therapies should be considered when a new diagnosis of heart failure is made in a young adult.
